# Genetic abnormalities in fetal congenital heart disease with aberrant right subclavian artery

**DOI:** 10.1038/s41598-022-20037-6

**Published:** 2022-09-23

**Authors:** Hairui Sun, Lu Han, Xiaoyan Hao, Zhaoyi Chen, Jingyi Wang, Tong Yi, Xiaoxue Zhou, Xiaoyan Gu, Jiancheng Han, Ye Zhang, Lin Sun, Xiaowei Liu, Siyao Zhang, Yong Guo, Hongjia Zhang, Yihua He

**Affiliations:** 1 Department of Echocardiography, Beijing Anzhen Hospital, Capital Medical University, No. 2, Anzhen Road, Chaoyang District, Beijing, 100029 China; 2 Department of Cardiac Surgery, Beijing Chao-Yang Hospital, Capital Medical University, Beijing, China; 3 Department of Cardiac Surgery, Beijing Anzhen Hospital, Capital Medical University, No. 2, Anzhen Road, Chaoyang District, Beijing, 100029 China

**Keywords:** Clinical genetics, Disease genetics

## Abstract

Fetal aberrant right subclavian artery (ARSA) is a relatively common sonographic finding. Congenital heart disease (CHD) is the most common structural abnormality in patients with ARSA. We aimed to assess the prevalence of genetic abnormalities, particularly sequence variants, in fetuses with CHD and ARSA. By clinical phenotyping and genomic sequencing, we retrospectively reviewed all fetuses with a prenatal diagnosis of CHD combined with ARSA at a single center. As a result, we identified 30 fetuses with ARSA combined with CHD, with conotruncal anomalies being the most common (n = 12, 40%), followed by left ventricular outflow tract obstruction (n = 6, 20%) and atrioventricular septal defects (n = 6, 20%). Overall, 18 (60%) cases had a genetic diagnosis. Copy number variation sequencing analysis identified six (20%) fetuses with aneuploidy and seven (23%) with pathogenic copy-number variants. Whole-exome sequencing (WES) analysis of the remaining 17 cases revealed diagnostic genetic variants in five (29%) cases, indicating that the diagnostic yield of WES for the entire cohort was 17% (5/30). Our findings reveal the high burden of genetic abnormalities in fetal CHD with ARSA. Single-gene disorders contribute substantially to the genetic etiology of fetal CHD with ARSA.

## Introduction

Aberrant right subclavian artery (ARSA) is the most common aortic branching variant. In the left aortic arch with ARSA, the right subclavian artery arises independently from the descending aorta instead of its typical origin from the brachiocephalic trunk^1^. The incidence of ARSA in the general population is estimated to be 1.2% postnatally and 0.6–1.5% prenatally^1,2^.

Prenatal demonstration of ARSA raises a concern for genetic aberrations. The association between ARSA and genetic abnormalities in the general population has been widely studied and reported. Most studies found that isolated ARSA probably had no clinical significance and did not serve as a marker for an invasive prenatal chromosomal test^3–6^. However, the genetic testing used in these studies, including karyotyping and chromosomal microarray analysis, can only identify chromosomal abnormalities and cannot detect single-gene disorders. The contribution of single-gene disorders to ARSA is unknown but is potentially substantial.

On the other hand, although ARSA is associated with congenital heart disease (CHD), with a significantly higher incidence in the CHD population than in the general population^1,7^, literature on the comprehensive assessment of genetic abnormalities in fetal CHD with ARSA is lacking. In particular, no data exist regarding the application of exome sequencing to the prenatal phenotype of coexistence of CHD with ARSA.

CHD is the most common congenital defect worldwide, occurs in 1% of live births, and is associated with significantly high perinatal morbidity and mortality^8,9^. Identifying a genetic diagnosis of fetal CHD can inform prenatal management, optimise post-natal outcomes, and aid in the counselling of parents in both index and subsequent pregnancies^10,11^.

This study aims to estimate the prevalence of genetic abnormalities in fetuses with ARSA and CHDs, particularly the contribution of single-gene disorders, by assessing results from genomic sequencing, including copy number variation sequencing (CNV-seq) and whole-exome sequencing (WES). This study will also evaluate the potential diagnostic yield of WES for fetal CHD with ARSA.

## Methods

### Participant recruitment

We retrospectively reviewed our experience with fetuses diagnosed with ARSA coexisting with other congenital heart defects at Beijing Anzhen Hospital, Capital Medical University, from July 2015 and July 2020. Routine fetal ultrasound anatomy scans were performed for pregnant women. If CHD was suspected, echocardiography was subsequently performed in Beijing Anzhen Hospital, a regional and national referral center. Parents who opted for terminating their pregnancies and genetic testing were offered participation in this study. Samples of father-mother-fetus trios were collected for sequencing. Prenatal medical records regarding gestational age at diagnosis, gender, family history, cardiac and extracardiac abnormalities, and the results of genetic testing were collected.

### Fetal ultrasound and echocardiography examination

All ultrasound examinations were performed by experienced operators using the General Electric Voluson E8 ultrasound system with transabdominal 2- to 4-MHz curvilinear transducers (GE Healthcare Ultrasound, Milwaukee, WI, USA) or the Aloka SSD ultrasound system (Aloka, Tokyo, Japan) using transabdominal 3- to 6-MHz curvilinear transducers. A complete fetal echocardiographic examination, including twodimensional (2D), M-mode, color, and pulse Doppler echocardiography, was performed according to the American Society of Echocardiography guidelines and standards for performance of the fetal echocardiogram to ascertain the presence of CHD^12^.

### Copy-number variation sequencing

Both CNV-seq and WES were performed as described previously^13,14^. We performed CNV-seq as follows. RNA-free genomic DNA isolated from the umbilical cord was used for library construction. The DNA library was sequenced on Illumina Hiseq 4000 or Illumina Novaseq. Burrows-Wheeler Aligner^15^ (BWA) was used to compare and analyze the sequence reading information with the human reference genome (hg19/GRCh37) to obtain the bioinformatics results and determine the existence of chromosomal aneuploidy variation and CNVs. According to the American College of Medical Genetics (ACMG) standards and guidelines for interpretation of CNVs, CNVs were classified into five categories: pathogenic, likely pathogenic, uncertain significance, likely benign, or benign^16^.

### Whole-exome sequencing

WES was performed on DNA extracted from the fetal umbilical cord or parental blood^13,14^. The captured library was sequenced on Illumina Hiseq 4000 or Illumina Novaseq. The sequencing reads were aligned to the NCBI human reference genome (hg19/GRCh37) using BWA. Variant calling and quality filtering of variants were performed with Genome-Analysis-Toolkit^17^. ANNOVAR was used for annotation of genes and functional variant effects^18^. Pathogenicity of variants was determined according to current ACMG guidelines^19^. Variants classified as pathogenic or likely pathogenic were considered positive genetic results. Sanger sequencing was used to validate the presence of positive genetic results.

### Statistical analysis

Categorical variables are presented as frequencies (percentage) and were compared using the Pearson χ^2^ test or Fisher’s exact test. Statistical analysis was performed using SPSS version 23 (SPSS, Chicago, IL, USA). Two-sided P values < 0.01 were considered significant.

### Ethical approval

This retrospective study was approved by the institutional review board of the Medical Ethics Committee of Beijing AnZhen Hospital. All parents agreed to participate in this study and provided signed informed consent.

## Results

### Cohort characteristics

The demographics, cardiac and extra-cardiac characteristics, and sequencing information of the entire cohort are summarized in Table 1. This cohort was composed of 14 males and 16 females. The median maternal age was 29 (range 20–38) years, and the fetuses were assessed at a median gestational age of 24 (range 16–29) weeks. Twelve (40%) cases had ultrasound-detected extra-cardiac malformations and 18 (60%) were isolated CHD. Most of our CHD patients had a complex disease consisting of more than one distinct anomaly. Patients were categorized to the single-most relevant anomaly to limit overlap between groups. The commonest CHD in this study was conotruncal anomalies, followed by left ventricular outflow tract obstruction (LVOTO) then atrioventricular septal defects (AVSD) (Table 1).

**Table 1:** General information of the entire cohort.

**Cardiac diagnoses (n = 30)**	
Conotruncal anomalies	12 (40.0%)
LVOTO	6 (20.0%)
AVSD	6 (20.0%)
APVC	2 (6.7%)
Heterotaxy	2 (6.7%)
Others	2 (6.7%)
**Extra-cardiac malformations**	
Yes	12 (40.0%)
No	18 (60.0%)
**Gender**	
Male/female ratio	0.875:1
Male	14 (46.7%)
Female	16 (53.3%)
**Maternal age (years)**	
21–40, median:29	
**Gestational age (weeks)**	
16–39, median:24	
**Sequencing information**	
CNV-seq	30
WES	17

*APVC* anomalous pulmonary venous connection, *AVSD* atrioventricular septal defect, *CNV-seq* copy-number variation sequencing, *LVOTO* left ventricular outflow tract obstruction, *WES* whole-exome sequencing.

### Genetic results

**Figure 1:**
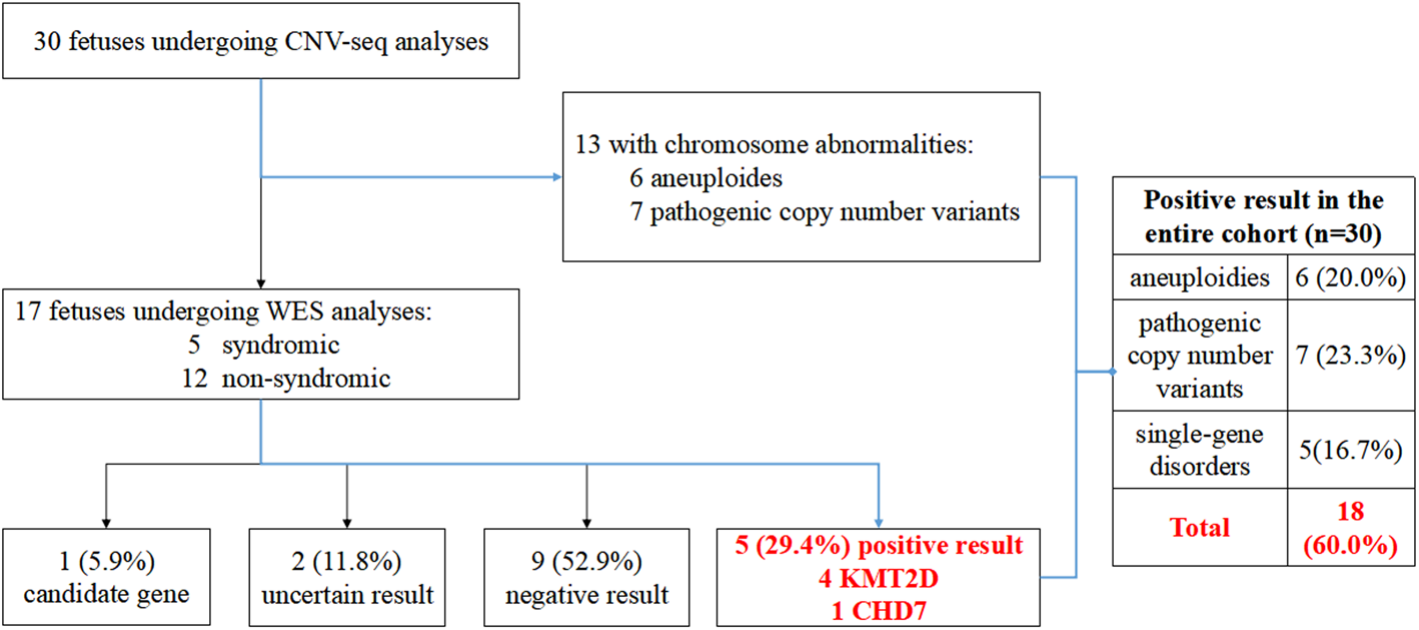
Flowchart showing the genetic testing and findings of next-generation sequencing analysis in the study of fetal congenital heart disease with aberrant right subclavian artery. In this cohort, ‘syndromic’ and ‘non-syndromic’ refer to presence and absence, respectively, of fetal structural abnormalities detected on ultrasound. *CNV* copy-number variant, *CNV-seq* CNV sequencing, *WES* whole-exome sequencing.

The flowchart of genetic analysis progression is shown in Fig. 1. Overall, a genetic cause was identified in 18(60%) cases, comprising six (20%) cases with aneuploidies, seven (23%) cases with pathogenic CNVs, and five (17%) cases with sequence variants, respectively. The genotype and phenotype information of the 18 cases are shown in Tables 2, 3 and 4. Four of the six aneuploidy cases were trisomy 21 (T21), of which three had conotruncal anomalies (Table 2). Among the seven cases with pathogenic CNVs, four were 22q11.2 deletion syndrome (22q11.2DS), and the corresponding cardiac phenotype was all conotruncal anomaly (Table 3). In addition, there were two cases with unbalanced translocation whose parents were balanced translocation carriers. We further found the diagnostic sequence variants in five cases through WES analysis (Table 4), accounting for 29% of those without chromosome abnormalities and 17% of the entire cohort. Among the five cases of sequence variation, four cases occurred in the KMT2D gene.

**Table 2:** Aneuploidies in the cohort.

ID	CHDS	Extracardiac malformation	Aneuploidy
11	AVSD	Absent nasal bone	Trisomy 21
12	AVSD	None	Trisomy 21
20	AVSD	Absent nasal bone, duodenal atresia, AMP5F	Trisomy 21
23	CA	Cleft palate	Trisomy 21
19	LVOTO	None	Trisomy 13
27	APVC	Hallux valgus, oligodactyly, abnormal lung lobation	Trisomy 18

*AMP5F* abnormality of the middle phalanx of the 5th finger, *APVC* anomalous pulmonary venous connection, *AVSD* atrioventricular septal defect, *CA* conotruncal anomalies, *CHDS* congenital heart defect subtype, *LVOTO* left ventricular outflow tract obstruction.

**Table 3:** Pathogenic copy number variants in the cohort.

ID	CHDS	Extracardiac malformation	Copy number variant	Size (Mb)
3	CA	None	seq[hg19]del(22)(q11.21)chr22:g.18910000_21464000del	2.55
7	CA	Aplasia of the thymus, small placenta, oligohydramnios	seq[hg19]del(22)(q11.21)chr22:g.18911000_21464000del	2.55
16	CA	None	seq[hg19]del(22)(q11.21)chr22:g.18880000_21470000del	2.59
30	CA	None	seq[hg19]del(22)(q11.21)chr22:g.19025000_21460000del	2.43
21	CA	Single umbilical artery	seq[hg19]del(8)(p21.3p21.2)chr8:g.22794000_24092000del	1.30
15	AVSD	None	seq[hg19]del(1)(q42.3q44)chr1:g.236290000_249225000del	12.94
			seq[hg19]dup(21)(q22.13q22.3)chr1:g.39620000_48105000dup	8.48
22	TH	Arachnoid cyst	seq[hg19]del(6)(p25.3p25.2)chr6:g.120000_2515000del	2.40
			seq[hg19]dup(2)(p25.3p25.1)chr2:g.1_11150000dup	11.15

*AVSD* atrioventricular septal defect, *CA* conotruncal anomalies, *CHDS* congenital heart defect subtype, *TH* Tricuspid hypoplasia.

**Table 4:** Clinical phenotypes and identified pathogenic variants in cases with positive results.

ID	CHDS	Extracardiac malformation	Gene	Variant	Zygosity	Parental origin	Pathogenicity
1	CA	None	KMT2D	NM_003482.3:c.9967_9970delCCAG,p.Pro3323Valfs*6	Het	De novo	Pathogenic
8	AVSD	None	KMT2D	NM_003482.3:c.8074_8075delCG,p.Arg2692Alafs*31	Het	De novo	Pathogenic
24	LVOTO	None	KMT2D	NM_003482.3:c.8159G>A,p.Trp2720*	Het	De novo	Pathogenic
28	CA	None	KMT2D	NM_003482.3:c.1116_1117delTT,p.Ser373Thrfs*10	Het	De novo	Pathogenic
10	SP	Cleft lip and palate, alveolar ridge cleft	CHD7	NM_017780.3:c.5405-17G>A	Het	De novo	Pathogenic

*AVSD* atrioventricular septal defect, *CA* conotruncal anomalies, *CHDS* congenital heart defect subtype, *Het* heterozygous, *LVOTO* left ventricular outflow tract obstruction, *SD* septal defects.

When considering the associated heart defects with ARSA, the prevalence of a genetic diagnosis was highest in AVSD (5/6, 83%), followed by conotruncal defects (8/12, 67%) then LVOTO (2/6, 33%). Genetic causes were identified more frequently in fetuses with extra-cardiac malformations (8/12; 66.7%) than in those without extra-cardiac malformations (10/18; 55.6%). However, the difference was not significant (Fisher's exact test; p = 0.7).

Besides the findings presented above, we also detected two uncertain significance variants and two novel candidate genes for CHD (Supplementary Table 1). All these variants met the following conditions: (1) were absent in the gnomAD database, (2) were LoF variants occurring in the LoF-intolerant genes (pLI score > 0.99)^20^ or non-synonymous variants predicted to be damaging by all silico predictions (SIFT, PolyPhen-2, MutationTaster). Further research is required to evaluate the pathogenicity of these variants.

## Discussion

This study represents the first cohort to assess the sequence variants in fetuses with CHD and ARSA. The diagnostic yield of 60% in our study is substantially higher than that of 10–30% in unselected CHD studies^21–24^, reflecting the significant burden of genetic abnormalities underlying CHD complicated with ARSA. Our findings show that single-gene disorders, especially KMT2D gene abnormality, also play an essential role in CHD combined with ARSA; AVSD and conotruncal anomalies are definite signs for genetic testing among the cardiac defects coexistence with ARSA. Its clinical implication is that prenatal WES should be performed to identify genetic diagnoses to facilitate perinatal decision-making and management when conventional tests (karyotype and microarray) are not diagnostic.

Our study's significant strengths are combining CNV-seq and WES to identify genetic diagnoses, including chromosomal abnormalities and sequence variants. In contrast, all previous studies used karyotype analysis or chromosomal microarray analysis, focusing only on chromosomal abnormalities^3,25^. This genetic testing strategy allowed us to reveal a more comprehensive genetic profile of ARSA. More importantly, we found for the first time that nearly 30% of patients without chromosomal abnormalities carried diagnostic sequence variants. Another strength of this study is that it focused on a specific phenotype, namely fetal CHD with ARSA, rather than unselected CHD or ARSA. While narrow in its focus, our approach is more effective and accurate in assessing the predictive value of ARSA for genetic abnormalities in fetuses with CHD. The yield of 60% in our series is substantially higher than the 10–30% yield in studies of unselected CHD^21–24^, suggesting that demonstration of ARSA in pregnancies with CHD may serve as a solid red flag for genetic abnormalities. Pregnancies with CHD and ARSA should be referred for invasive prenatal testing.

Although several recent studies have researched aneuploidy or 22q11.2DS in ARSA cases^3–6,25–28^, evidence on the prevalence of chromosome abnormalities and sequence variants in ARSA with specific heart defects is limited. In our study, the commonest cardiac phenotype associated with ARSA was conotruncal anomalies, followed by LVOTO and AVSD. This association is in line with the previous literature^1^.

The prevalence of genetic causes varied significantly between CHD subgroups. The AVSD group was most associated with a genetic diagnosis, and the total positive rate was 83% (5/6), among which T21 was the most common. The conotruncal defects group was also frequently accompanied by a genetic diagnosis, of which 22q11.2DS is the most common. On the other hand, genetic diagnoses were rarely encountered among ARSA fetuses with a diagnosis of LVOTO. On the other hand, in terms of genotype, the most common chromosomal abnormalities in this study were T21 and 22q11.2DS, respectively. The association between chromosomal abnormalities, especially T21 and 22q11.2DS, and CHD is well documented in the literature^25^. This study also identified two fetuses with unbalanced chromosomal rearrangement and confirmed their parents to be carriers of a balanced translocation. Notably, the offspring of balanced translocation carriers are at high risk of unbalanced rearrangement, and they should consider assisted reproduction.

The prevalence of sequence variants (29.4%) was higher in our fetal CHD cohort than in the previous fetal CHD cohort. Several studies have reported diagnostic rates of sequence variants in fetal CHD ranging from 5.2 to 13.5%^24,29–31^. This difference reflects the population selection bias, as we only included fetal CHD with ARSA, while other studies included all cases with and without ARSA. Therefore, the estimated diagnostic yield in our cohort may be more accurate and more suitable for prenatal counseling for fetal CHD associated with ARSA. The coexistence of ARSA in fetal CHD can be a crucial anatomic marker for genetic diagnosis. Another interesting finding was that four of the five sequence variants occurred in KMT2D, the causative gene of Kabuki syndrome-1 (OMIM:147920). This high proportion of KMT2D mutations may reflect the high incidence of KMT2D mutations in CHD because an increasing number of exome sequencing studies have shown that KMT2D is the most common mutated gene in CHD^14,32^. More importantly, this finding suggests that ARSA may be an indicator of Kabuki syndrome. However, more evidence is needed to support this hypothesis.

### Limitations

Major limitations of the study include the small sample size, its retrospective observational nature, and the absence of a control group with fetal CHD without ARSA. We acknowledge that there may be a selection bias because this is a small series study from a tertiary hospital. Our center is one of the major referral hospitals for fetal CHD. Therefore the most severe cardiac malformation, such as conotruncal anomalies and AVSD, may be found more frequently.

## Conclusions

In conclusion, we used CNV-SEq and WES to comprehensively investigate genetic abnormalities, including aneuploidy, CNV, and single-gene disorder, in a cohort of fetal CHD with ARSA. Our results indicate a high burden of genetic abnormalities in fetal CHD with ARSA and suggest that ARSA in pregnancies with CHD may serve as a solid red flag for genetic abnormalities. A single-gene disorder diagnosis would not have been found in about 30% of cases without chromosome abnormalities if WES had not been performed. In genotype-phenotypic association, KMT2D is the most frequently mutated gene, while AVSD and conotruncal anomalies are most commonly associated with genetic abnormalities. However, future research, which offers WES in prospective extensive cohort studies on fetal CHD with ARSA, is needed to obtain more reliable estimates.

## Supplementary Information

Supplementary Table 1.

## Data Availability

All datasets generated for this study are included in the article/Supplementary Material. This study is compliant with the “Guidance of the Ministry of Science and Technology (MOST) for the Review and Approval of Human Genetic Resources”, which requires formal approval for the export of human genetic material or data from China. The phenotypic data that support the findings of this study are available from the corresponding author upon reasonable request.
